# Laser Shock Peening on Zr-based Bulk Metallic Glass and Its Effect on Plasticity: Experiment and Modeling

**DOI:** 10.1038/srep10789

**Published:** 2015-05-20

**Authors:** Yunfeng Cao, Xie Xie, James Antonaglia, Bartlomiej Winiarski, Gongyao Wang, Yung C. Shin, Philip J. Withers, Karin A. Dahmen, Peter K. Liaw

**Affiliations:** 1 Center for Laser-based Manufacturing, School of Mechanical Engineering, Purdue University, West Lafayette, IN 47907, USA; 2 Department of Materials Sci. & Eng., The University of Tennessee, Knoxville, TN 37996, USA; 3 Department of Physics, University of Illinois at Urbana Champaign, IL 61801, USA; 4 School of Materials, The University of Manchester, Grosvenor Street, Manchester, M13 9PL, U.K

## Abstract

The Zr-based bulk metallic glasses (BMGs) are a new family of attractive materials with good glass-forming ability and excellent mechanical properties, such as high strength and good wear resistance, which make them candidates for structural and biomedical materials. Although the mechanical behavior of BMGs has been widely investigated, their deformation mechanisms are still poorly understood. In particular, their poor ductility significantly impedes their industrial application. In the present work, we show that the ductility of Zr-based BMGs with nearly zero plasticity is improved by a laser shock peening technique. Moreover, we map the distribution of laser-induced residual stresses via the micro-slot cutting method, and then predict them using a three-dimensional finite-element method coupled with a confined plasma model. Reasonable agreement is achieved between the experimental and modeling results. The analyses of serrated flows reveal plentiful and useful information of the underlying deformation process. Our work provides an easy and effective way to extend the ductility of intrinsically-brittle BMGs, opening up wider applications of these materials.

Since bulk metallic glasses (BMGs) were developed^1^,^2^,^3^,^4^,^5^,^6^,^7^,^8^,^9^, significant efforts have been made to study their plastic-deformation behavior under different loading modes, including nanoindentation, compression, and bending experiments^10^,^11^,^12^. The plastic flow in metallic glasses is widely considered to be related to the formation of localized shear bands. In BMG samples loaded in uniaxial tension, crack initiation and propagation occur almost immediately after the formation of the first shear band^13^. As a result, BMGs show essentially zero ductility in tension and limited plasticity in compression^14^.

It was recently reported that the ductility of the Vitreloy 1 (Vit-1) BMG [Zr_41.25_Cu_12.5_Ni_10_Ti_13.75_Be_22.5_, atomic percent (at.%)] can be improved by controlling residual stresses^14^. Shot-peened BMGs show the increased plasticity in bending and compression due to the reduced likelihood of surface cracking and more homogeneous deformation induced by a high population of shear bands^14^. Compared with conventional shot peening, which introduces residual stresses into distances of the order of hundreds of microns^15^, the laser shock peening (LSP) process is capable of introducing residual stresses to much greater depths (millimeters) in metals and other materials^16^,^17^. Several research activities have been done on metallic glasses using the laser-surface treatment^18^,^19^,^20^,^21^. To enhance mechanical properties, laser treatment can introduce crystalline phases and make the glassy alloys into BMG composites. For instance, Tariq *et al.* found that the surface hardness of BMGs can be altered by crystalline particles formed during the laser-pulse irradiation^18^. Fornell *et al.* reported that hardness obtained in nanoindentation tests can be tuned by controlling the extent of the induced crystallization using different laser intensities^19^. Wu *et al.* found that the plastic strain of the CuZr-based BMG is prolonged by the laser-surface treatment through embedding micro- or nano-crystals^20^. Besides the above three cases, laser-surface melting, along with the helium jet flow at high cooling rates, was reported to be capable of improving the compressive plasticity of BMGs without crystallization^21^. In the present study, we report a LSP process with water confinement and Al-coating to introduce compressive residual stresses into the BMG material, so as to investigate the extent to which this process can improve the plasticity of BMGs without the introduction of crystalline phases under compression testing. Our work provides an easy and effective way to prolong the ductility of intrinsically-brittle BMGs, followed by a series of advanced experimental characterizations, theoretical analyses, and modeling. Besides, the LSP process could introduce large magnitudes of residual stresses and leave a smooth surface, and can be automated and fully-developed in industry. By taking these advantages, the present work is expected to speed up the commercialization of BMGs, broaden their engineering applications, and eventually improve our daily life with their unique properties.

In the LSP process (Figure S1, experimental details in the Methods section), a high-energy laser discontinuously irradiates the target surface, generating a high-pressure plasma shock wave each pulse, which is concentrated into the sample by the water confinement^22^,^23^. When the pressure wave propagates into the substrate material as a shock wave, compressive residual stresses can be introduced deep into the surface region^24^, which, in turn, can improve the material’s fatigue behavior and wear resistance. To protect the substrate from the thermal damage that may occur during the LSP process and to improve the absorption of laser energy, an Al-coating layer is usually applied to the surface.

Given that the stress measurement on BMGs by x-ray diffraction is not as feasible and precise as on crystalline materials, a focused-ion-beam (FIB) micro-slot cutting (μSC) technique was used to measure the residual stress of the laser-peened BMG, having a spatial resolution of tenths of a micron^25^,^26^,^27^. Using the scheme shown in Fig. 1a, the residual-stress distribution was measured on the laser-peened Vit-105 BMG sample, Zr_52.5_Cu_17.9_Ni_14.6_Al_10.0_Ti_5.0_ (at.%), and on a face perpendicular to the peened surface. To map the stresses, a series of micro-slots of 15 × 2 × 0.4 μm^3^ in size were made on the specimen surface (Fig. 1b) using the FIB of a dual-beam Field Emission Gun Scanning Electron Microscope / Focused Ion Beam (FEGSEM/FIB) instrument^25^. In order to measure the displacement field caused by each microslot, a pattern of nano Pt dots was applied locally by the FIB-assisted deposition (Fig. 1c)^26^. The deformation fields in the vicinity of slots were, then, reconstructed by the digital image correlation (DIC) of FEGSEM photos recorded during milling (Fig. 1d). Since each slot has a wedge shape and a finite length, the residual stresses are inferred by fitting a reference displacement field obtained from the finite-element model (FEM) with the recorded displacement field^25^. In this way, residual-stress distributions have been characterized as a function of the distance from the laser-peened surface to a depth of 1,200 μm with a spatial resolution of 30 μm (arising from the spacing between the slots see Fig. 1c). Residual stresses were measured in this way for the as-cast BMG, after mechanical polishing and after laser peening.

To calculate the propagation of the shock wave into the sample and thereby to predict the residual-stress distribution on the target material, a new three-dimensional (3-D) FEM has been developed (details in the Supplementary Material). The FEM-calculation procedure of residual stresses is explained in Figure S4. The plasma pressure and other parameters are put into ABAQUS/Explicit first, and calculations are performed until the saturation of the plastic deformation has occurred in the target. The calculation in the ABAQUS/Explicit is then stopped, and the deformed body with all the stress, strain, and displacement states is imported into the ABAQUS/Standard to determine the residual-stress field at a state of static equilibrium. For a multi-shock overlapping LSP process, the residual stress and strain states from the first impact become the initial stress and strain states of the material for the second impact. The material state is imported back into ABAQUS/Explicit, and then the analysis procedure is repeated^28^,^29^. Within this procedure, both the single shot and overlapping LSP can be handled successfully. The computational cost is also reduced significantly by combining the ABAQUS/Explicit and ABAQUS/Standard^24^. Then the experimentally-measured residual-stress data and model-predicted results were compared. In addition, the serrated plastic flow regime in the stress-strain curve was analyzed^30^, and the statistical difference between compression results of the laser-treated and as-cast samples was extracted.

## Results

**Figure 2:**
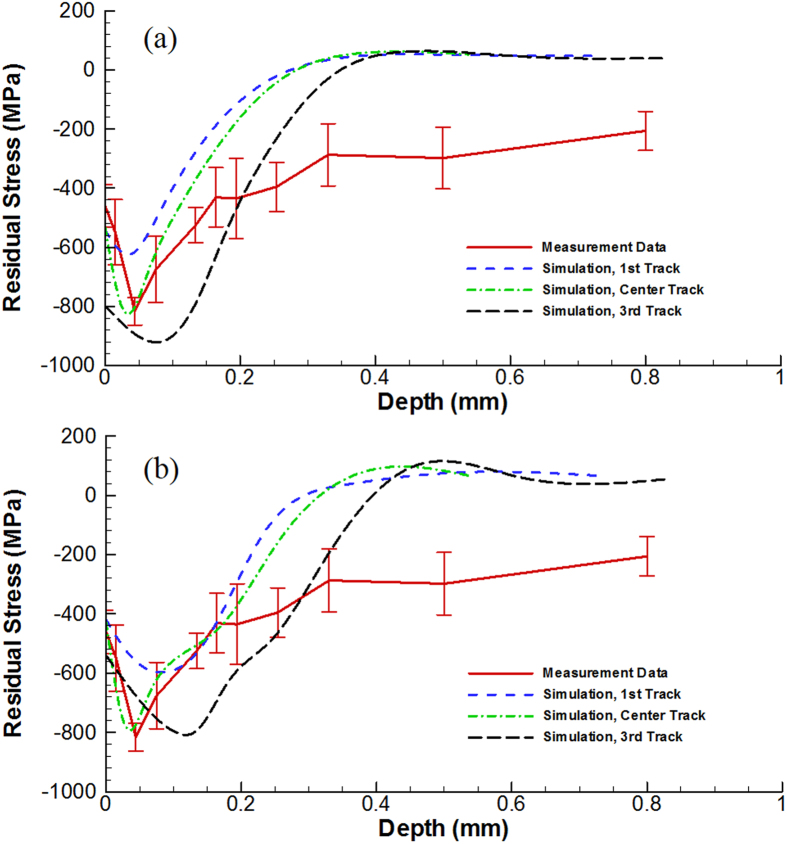
Residual stresses for the bulk metallic glass (BMG) sample (Vit-105) after LSP to a power density of 8.64 GW/cm compared to model simulations, (**a**) assuming that there is no strain-rate effect, and (**b**) including strain-rate softening at high strain rates. The compressive plateau is taken to be the residual-stress state introduced by the surface preparation prior to micro-slotting. The colors of simulated tracks correspond to those in Figure 1a.

The variations in the measured residual stresses after laser peening as a function of depth are described in Fig. 2 (the red solid line). The peak compressive stress (810 MPa) occurs at a depth of about 50 μm. Note that the residual stress for a small sample size could be lowered due to the lack of constraint, when compared to a thicker sample. It is also noteworthy that the stress reaches a plateau of −300 MPa at a depth of about 300 μm. Measurements on the as-cast samples show that the fabrication process gives rise to a compressive residual stress of −40 MPa near the surface, and that these stresses can rise to between −110 and −400 MPa after mechanically polishing. Consequently, it would appear that the residual stresses measured on the polished sides of the sample at distances greater than 300 μm from the peened surface are due to sample preparation.

In accordance with the conditions studied for the residual-stress measurement, a laser power density of 8.64 GW/cm^2^ was considered in the modelling efforts. The residual stresses predicted by our FEM are shown in Fig. 2a. The depth of the compressive residual stress zone after LSP is predicted to be around 300 μm, which is in good agreement with the effective depth of 300 μm measured experimentally. The predicted maximum residual stress of −830 MPa along the center track is very close to the maximum value of −820 MPa measured. Some differences can be observed for the residual stresses along different tracks due to the overlapping effect of the laser treatment.

**Figure 3:**
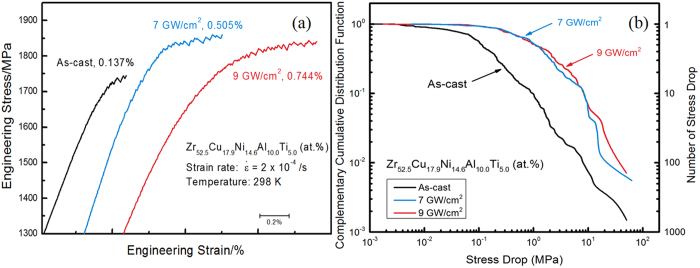
Compression testing results for as-cast and laser-treated BMG samples (**a**) stress-strain curve denoted by the laser power density and the plastic strain (all samples fractured at the right-end point of each curve, respectively), and (**b**) log-log plot of the complementary cumulative distribution (CCDF) of stress-drop sizes in the serration regime of the stress-strain curve

The compressive mechanical test results are shown in Fig. 3a for the peened and unpeened samples. It is evident that the plasticity and the fracture strength of the BMG sample are improved upon LSP. Note that a decrease in the slope of the curve with a laser power of 9 GW/cm^2^ may be caused by the deformation-induced softening in the laser-peened layer^14^,^31^. Taking a closer look at the serrated flow regime, the plastic strain of laser-treated samples is about 5 ~ 7 times greater than that of the as-cast sample. Even though the mechanism of the stress-flow serration in metallic glasses is still unclear, it is almost certainly connected to shear-band propagation^32^,^33^. The present result suggests that near-surface shear bands introduced by laser peening (Figure S2) along with the associated compressive residual stresses may impede the catastrophic propagation of macro-shear bands, allowing a more homogeneously-distributed network of shear bands to develop, which are beneficial for homogenizing plastic deformation.

Furthermore, we studied the serration regime in the stress-strain curve, and the complementary cumulative distribution function (CCDF) for stress drops, as described in Fig. 3b. Each stress drop represents a slip avalanche, and is caused by shear-band propagation. In terms of energy, shear-band propagation and arrest refer to periods of elastic energy release and accumulation, respectively. The serrations, or stress drops, in the stress-strain curves are seen, wherever the stress suddenly drops from a higher to a lower value. The CCDF, *C(S)*, of the stress-drop sizes *S*, gives the number of stress drops larger than or equal to a size *S*, *N(S)*, divided by the total number (*N*_*total*_) of stress drops observed in the experiment (see Reference 30 for more details), which can be expressed as:


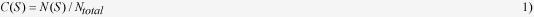


This definition implies that C(S) decays monotonically with C(S = 0) = 1 and C(S  = S_max_) = 1/N_total_, where S_max_ is the largest stress drop observed in the experiment. It is clear from Fig. 3b that the total number of slips (the reciprocal of the right end value in the CCDF curve, i.e., 670 for the as-cast case, 180 for 7 GW/cm^2^, and 140 for 9 GW/cm^2^) decreases for higher laser powers. Also in the as-cast brittle case, the range of slip sizes appears to extend down to smaller slip sizes than is observed for the laser-treated samples.

## Discussion

(1) Different from the previous work, a 100 μm-aluminum coating was applied to the surface to avoid the heat-induced nanocrystallization and hydration of the sample. When the laser pulse with a sufficient intensity irradiates the surface, the aluminum film vaporizes and forms high-pressure plasma shock waves. Because of the short energy-deposition time (around 6–20 ns), the diffusion of the thermal energy away from the interaction zone is limited to a couple of microns and is preferred to be less than the thickness of the aluminum coating (100 μm) to maintain protection. Therefore, the local temperature on the surface of the BMG is still around room temperature. Under this condition, there is no heat-induced nanocrystallization of the glassy structure. Meanwhile, the water could be super-heated, if the laser intensity is high enough, and the water breakdown could even happen, in which the water would be ionized, and a water breakdown plasma could be formed. However in the present work, the laser power density is less than 10 GW/cm^2^. The water breakdown threshold is around 30 W/cm^2^ for the case investigated^34^. In addition, the aluminum coating also plays as a protective layer between the glass and water. As a result, it is impossible for the diffusion of hydrogen into the glass.

(2) The previous work^14^ on the more inherently-ductile Vit-1 BMG found that shot peening improved the compressive plastic elongation from 6% (the as-cast sample) to 11% (after shot peening). Here the ductility of Vit-105 has been extended from 0.137% to 0.505% and 0.744% by laser peening at 7 GW/cm^2^ and 9 GW/cm^2^, respectively. The extent of plastic deformation tends to increase, as the laser-power density is increased from 7 GW/cm^2^ to 9 GW/cm^2^. Meanwhile, the fracture strengths are also higher rising from 1,745 MPa for the as-cast condition to 1,869 MPa and 1,834 MPa for 7 GW/cm^2^ and 9 GW/cm^2^, respectively.

(3) The CCDF plot of Fig. 3b clearly depicts the shear-banding process and the different deformation mechanisms in the as-cast and laser-treated samples. It is reasonable to expect that the laser-peening induced shear bands behave as pre-existing shear bands at the surface (Figure S2), and facilitate the initiation of further deformation at these locations in the compression tests. In other words, these shear bands accommodate the deformation and reduce the chance of shear-band initiation at other places. In contrast, for as-cast samples, new shear bands will nucleate at more places without the pre-existing shear bands. However, the primary shear band (along the fracture direction) will dominate during the deformation process, resulting in relatively short lifetimes for these new shear bands. Therefore, more small slips will be present for as-cast samples than that for laser-treated samples. This trend agrees with the results shown in Fig. 3b, where clearly the as-cast samples have more small slips than the laser-treated samples. Meanwhile, the laser-induced compressive residual stresses impede shear-band propagation under the uniaxial loading and, thus, extend the elastic energy-accumulation period prior to ‘plastic’ deformation by slip. The combination of the above factors contributes to the larger plasticity for laser-peened samples, again in agreement with Fig. 3b, which shows that the laser-treated samples have fewer slips that are on average larger than in the as-cast samples. This mechanism is, hence, confirmed by the results of Fig. 3b.

(4) It should be noted that the dynamic behavior of the coating and substrate material play an important role in the shock-wave propagation and development of residual stresses. In the LSP process, the typical strain rate can be as high as 10^7^ s^-1^. Thus, the dynamic yield strength of the coating material is significantly increased due to the strain-rate hardening effects introduced by LSP. Here we describe the dynamic behavior of the coating material by the Johnson-Cook model^35^:


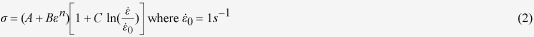


where σ is the flow stress, A = 120 MPa, B = 300 MPa, C = 0.1, and n = 0.35 are material constants for the aluminum coating^36^, *ε* is the plastic strain, 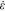 represents the strain rate, and 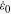 is the effective plastic strain rate of the quasi-static test used to determine the above materials constants. Previous work^37^ has shown that the peak yield stress varies slightly with the strain rate up to 1,000 s^-1^ at low temperatures (at least 295 K and 473 K) for the Zr-based Vit-1 BMG, which has a similar chemical composition to Vit-105. From the thermodynamic aspect, typically for LSP, the coating layer (e.g., Al) is ablated, preventing a significant increase in the temperature of the substrate, hence, thermal effects are neglected in our model. Thus, the substrate (e.g., Vit-105 BMG) can be modeled as an elastic-plastic material without a significant strain-rate effect.

(5) In order to assess the effect of strain rate on yielding and, hence, the laser-peened residual-stress state, we estimate the stress-strain curve based on the results^38^ up to a strain rate of ~ 5,000 /s, since there is no available data for the strain rate as high as 10^7^/s. The residual stresses after LSP were recalculated, using this strain-rate-softening effect, and the results were presented in Fig. 2b. The predicted depth of the compressive residual-stress zone after LSP is increased from around 300 to 450 μm (relative to the static case, Fig. 2a) depending on the LSP track due to the strain-rate softening effect^38^. Apart from this feature, the predictions are similar to those for the static case, for example, in predicting a maximum stress of −800 MPa between 50 and 100 μm from the surface (Fig. 2b).

(6) When considering the predictions, it should be remembered that the constitutive model of the BMG material in the present work may not be sufficiently accurate because the high strain-rate experimental data are not available. The available experimental data run out at 5,000/s, which is far less than the maximum strain rates typically observed in LSP (~ 10^7^/s). Therefore, the strain-rate softening behavior of BMGs under LSP may be underestimated. As indicated in Refs. 38-41, 39, 40, 41, the temperature rise inside the shear band may introduce strain softening at high strain rates, which may further increase the depth of the compressive region after LSP. Above all, the present study provides novel methods to improve the plasticity and strength of BMGs through both experimental and theoretical modeling effects. The combined experimental and theoretical strategy of laser treatments can open up wide opportunities to process BMGs with desired properties for applications.

In summary, residual stresses and local plastic deformation were successfully introduced into the Zr-based BMG sample by LSP, as demonstrated by the residual-stress measurements and modeling efforts. Increases in plasticity and strength were observed in the BMG sample and are probably due to the presence of compressive residual stresses and the formation of localized shear bands. It’s anticipated that these approaches could profitably be used to delay the stress at which the plastic flow commences and, thus, improves the ductility of BMGs.

## Methods

### Sample preparation

The Zr-based BMG Vit-105 (Zr_52.5_Cu_17.9_Ni_14.6_Al_10.0_Ti_5.0_ in atomic percent, at.%) was prepared by arc-melting mixtures of pure Zr, Cu, Ni, Al, and Ti metals in an argon atmosphere. The specimen is suction cast and cut into rectangular bars with dimensions of 4 × 2 × 2 mm^3^. After carefully polishing, four lateral surfaces (4 × 2 mm^2^) were coated with a 100 μm-thick aluminum tape.

### Laser shock peening

The 4 × 2 × 2 mm BMG sample was coated with an ablative Al tape before being placed into a water tank and shock peened using a laser wavelength of 1,064 nm and pulse duration of 6 ns with the power densities from 5 GW/cm^2^ to 10 GW/cm^2^. The laser-beam diameter was set to about 1.25 mm, and the overlap ratio was selected to be 50%. Three consecutive tracks of peening were applied to the surface with a distance of 0.625 mm between track centerlines. In this study, specimens with one treated surface are used for residual-stress measurements and for comparison with model predictions, while all four surfaces were laser peened for the compression tests. Note that four-side peening may introduce the multi-axial residual stress by the shock wave on the measured surface and complicate the measurement. So one-side peening is performed to show a clear trend of the depth profile of residual stresses introduced by laser.

### Compression experiments

The tests were conducted at a strain rate of 2 × 10^–4^ /s and room temperature, using a Material Testing System (MTS) servohydraulic-testing machine controlled by a computer. Three groups of samples were tested, which are as-cast, treated by laser power densities of 7.0 GW/cm^2^ and 9.0 GW/cm^2^, respectively. For each sample, the four lateral sides were treated with the same power density.

## Author Contributions

Y.C. and Y.C.S. conducted the laser treatment and developed the 3-D finite-element model. X.X., G.W. and P.K.L. fabricated the samples and conducted the compression experiments. J.A. and K.A.D. performed the serration analysis. B.W. and P.J.W. conducted the mapping and analysis of the residual-stress distribution. All authors contributed to the manuscript.

## Additional Information

**How to cite this article**: Cao, Y. *et al.* Laser Shock Peening on Zr-based Bulk Metallic Glass and Its Effect on Plasticity: Experiment and Modeling. *Sci. Rep.* **5**, 10789; doi: 10.1038/srep10789 (2015).

## Supplementary Material

Supplementary Information

## Figures and Tables

**Figure 1:**
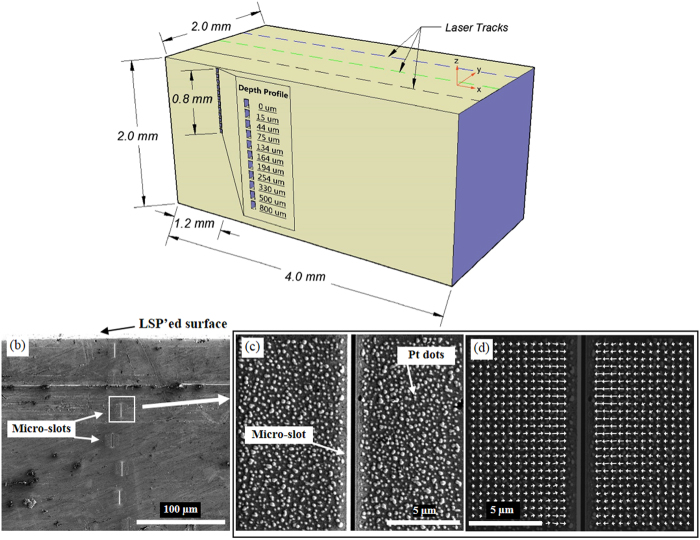
The micro-slot cutting method. (**a**) Schematic of residual-stress measurements on the side of the sample laser shock peened on the top surface, (**b**) Scanning electron microscopy (SEM) image showing a series of micro-slots introduced into the side of the specimen, (**c**) SEM image showing the random Pt-dot pattern deposited in the vicinity of a 0.4 μm-wide micro-slot, and (**d**) a displacement field (indicated by arrows proportional to the deformation) inferred by the digital image correlation (DIC) analysis.
