# Supplementary material for: Laser Shock Peening on Zr-based Bulk Metallic Glass and Its Effect on Plasticity: Experiment and Modeling
Source: Sci Rep. 2015 May 20;5:10789. doi: 10.1038/srep10789 (PMC4438488; doi:10.1038/srep10789)
Supplement: Supplementary Information [file srep10789-s1.doc]

**Laser Shock Peening on Zr-based Bulk Metallic Glass and Its Effect on Plasticity: Experiment and Modeling**

Yunfeng Cao^1^, Xie Xie^2^, James Antonaglia^3^, Bartlomiej Winiarski^4^, Gongyao Wang^2^, Yung C. Shin^1^, Philip J. Withers^4^, Karin A. Dahmen^3^, and Peter K. Liaw^2^

*^1^Center for Laser-based Manufacturing, School of Mechanical Engineering, Purdue University, West Lafayette, IN 47907, USA*

*^2^Department of Materials Sci. & Eng., The University of Tennessee, Knoxville, TN 37996, USA*

*^3^Department of Physics, University of Illinois at Urbana Champaign, IL 61801, USA*

*^4^School of Materials, The University of Manchester, Grosvenor Street, Manchester, M13 9PL, U.K.*

Nanosecond

laser

Half-wave

plate

Polarizer

Beam bump

Mirrors

Lens

Water tank

Linear motion stage

(x and y directions)

Workpiece

**-x**

**y**

Figure S1. Experimental setup for laser shock peening (LSP)


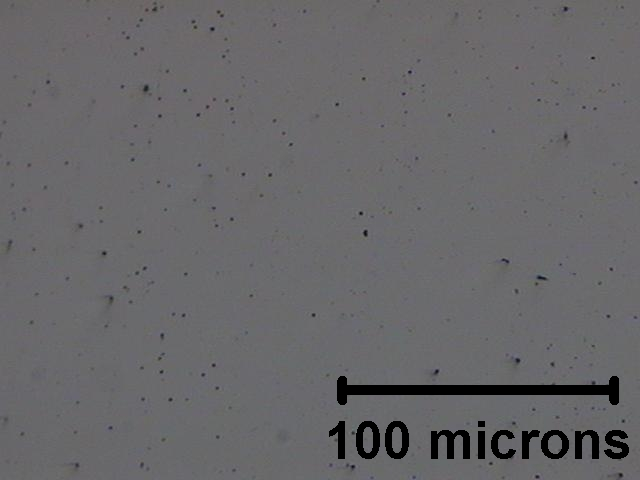

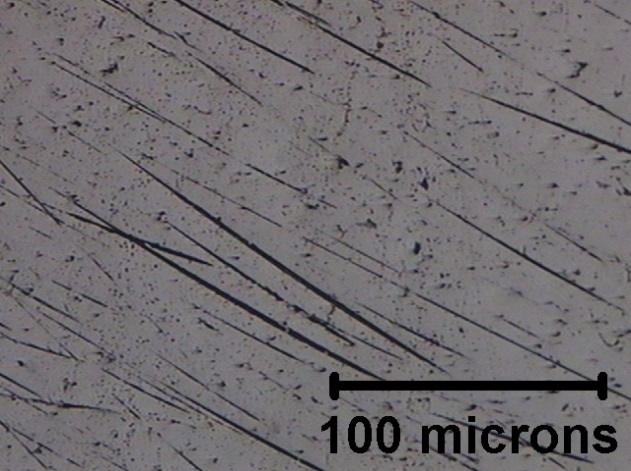


(a)

(b)


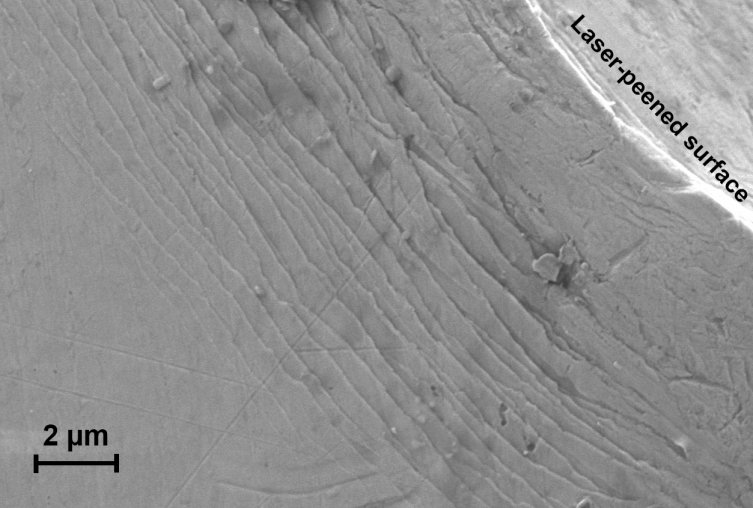


(c)

**Laser-induced shear bands**

Figure S2. Surface morphology of a bulk metallic glass (BMG) sample (a) before LSP (b) after LSP using a power density of 10.0 GW/cm^2^, and (c) a close-up showing the laser-induced shear bands on the lateral surface using a power density of 9.0 GW/cm^2^.


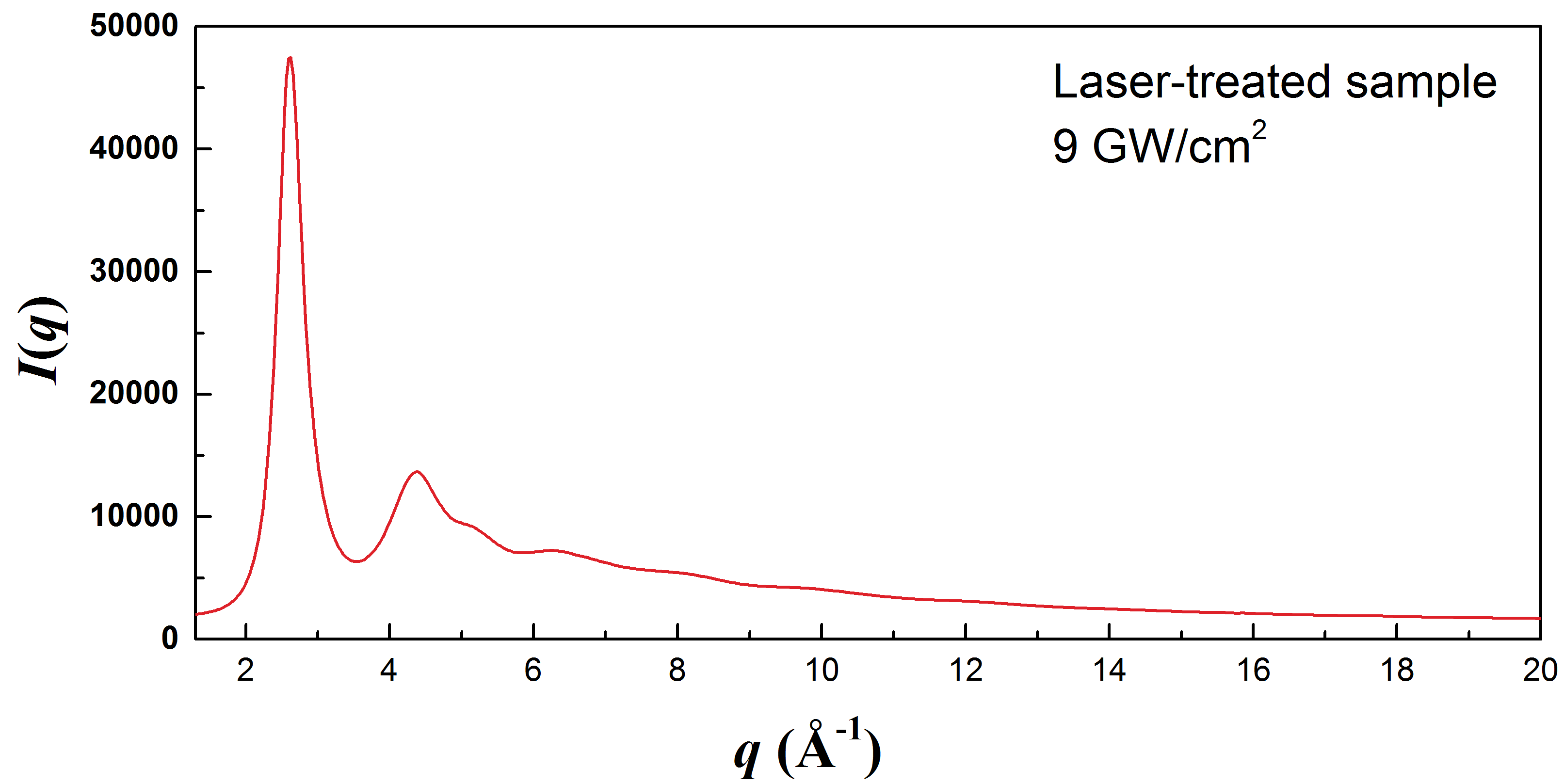


Figure S3. Synchrotron X-ray diffraction pattern of the laser-treated Zr_52.5_Cu_17.9_Ni_14.6_Al_10.0_Ti_5.0_ sample with a laser power of 9 GW/cm^2^.

Z

Coating

Water

Z = 0

Laser Radiation

(a) (b)

Figure S4. (a) Schematic diagram of the 1-D model setup, (b) Plasma-pressure history for laser shock peening of a BMG sample (Laser power density: 8.64 GW/cm^2^, laser wavelength: 1,064 nm, full width at half maximum (FWHM): 6 ns, coating: 100-µm Al tape, and substrate: Vit-105)

Confined Plasma Pressure, P(t), etc.

ABAQUS/Explicit, Dynamic Analysis

Calculation in ABAQUS/Explicit is performed until all the plastic deformation has occurred.

Data of stress, strain, displacement, etc.

ABAQUS/Implicit (Standard)

Static Analysis

Another LSP Impact?

Residual stress field, etc.

Analysis END

Yes

No

Figure S5. Finite-element modeling calculation procedure of residual stresses^S1,2^

Zr_41.25_Cu_12.5_Ni_10_Ti_13.75_Be_22.5_

Figure S6. Yield stress vs. strain rate for Vit-1 from dynamic-compression tests^S3^

Zr_52.5_Cu_17.9_Ni_14.6_Al_10.0_Ti_5.0_

Figure S7. Inferred strain-rate effects on the stress-strain curve for BMG (Vit-1)

**1. The Procedure of Laser Shock Peening**

The setup of laser shock peening (LSP) is shown in Figure S1 and details provided in the Methods section of the paper. The movement of the workpiece along the x and y directions was controlled by two linear motion stages. An Nd-Yttrium aluminum garnet (YAG) laser was used to generate a laser beam. Using this setup, the laser-power density and the beam size can be easily adjusted by fine-tuning the laser-beam path, the orientation of the half-wave plate, and the distance between the focus lens and the surface of the work-piece.

Figure S2 reveals changes of the surface morphology introduced by laser peening using optical microscopy and SEM. To maximize the laser-peening effect, we applied a relatively-higher laser power (10 GW/cm^2^). Compared to the smooth surface before the laser treatment, the peening introduced a certain extent of surface damage in the form of linear shear cracks/bands (see Figure S2), which is the direct result of the plastic deformation in the surface region. The presence of plastic deformation is due to the formation of localized shear bands. Concerning the observed change in plasticity, the formation of the localized shear bands is the fundamental contributing factor affecting plasticity. And the surface-roughening behavior might contribute to the change in some other mechanical properties, for example, the ultimate tensile strength. This surface-roughening phenomenon is similar to the observation of the surface of the BMG subjected to the high-speed impact^S4^. For the study described in the main text, we, therefore, reduced the laser power to 7 - 9 GW/cm^2^, such that the surface damage was much less severe, and shear bands can be found mainly in some regions below the edge of laser-peened surface, as shown in Figure S2c.

In addition, we performed synchrotron X-ray diffraction on the laser-treated samples at the beam line, 11-ID-C, of the Advanced Photon Source at the Argonne National Laboratory. As shown in Figure S3, no crystallization can be found in the diffraction pattern of the laser-treated sample with a laser power of 9 GW/cm^2^. This trend indicates that the aluminum coating is thick enough to prevent the nanocrystaliztion process from surface heating.

**2. Residual-Stress Measurements**

The measurements were taken at the slot locations shown in Figure 1a as a function of distance from the peened surface. The slots were oriented so as to measure the residual stresses along the longitudinal direction (the x direction) of the sample.

The slots were milled in a single increment to give the stress averaged over the removed slot depth (~ 2 microns). For an infinitely long, narrow slot, the surface displacements away from the slot, U_x_, are given by the following equation (see Ref. S5 for details).

$$U_{x}=\frac{2.243}{E'}\sigma_{x}\int_{0}^{a} \cos\theta\left(1+\frac{\sin^{2}\theta}{2\left(1-\nu\right)}\right)\times\left(1.12+0.18\sec h(\tan\theta)\right)da$$

where *x* is the distance from the slot, *v* is the Poisson’s ratio, $E'=E/(1-\nu^{2})$, *E* is the Young’s modulus, *σ_x_* is the residual stress, *a* is the slot depth, and $\theta=\arctan(x/a)$.

**3. Simple Analytical Model for Serration Behavior**

A simple mean-field theory (MFT) based model^S6-8^ was employed to quantitatively analyze the serrated plastic-flow behavior of the as-cast and laser-treated samples. The model assumes that weak spots exist in the material that can be triggered to slip, when the local stress exceeds a local failure stress under an external shear load. These weak spots are taken to be elastically coupled, which means that one slip will cause other slips and, consequently, lead to a slip avalanche. The material is sheared under a slowly-increasing stress or small strain-rate boundary conditions, which should be sufficiently slow to have a consecutive but discrete slip avalanche (not overlapped with each other). The elastic interaction between weak spots is sufficiently long-range to satisfy the assumption of infinite range interactions in MFT.

For a low imposed strain rate, Ω, the probability distribution function of the magnitude of stress drop, *S*, during the serration process can be predicted by MFT as:

$$D(S,\Omega)\sim S^{-\kappa}D'(S\Omega^{\lambda})$$

where *κ* and *λ* are exponents, with values of 1.5 and 2, respectively, in the MFT prediction for the steady state. *D*'(*S*Ω^λ^) is a universal scaling function.

The corresponding complementary cumulative distribution function (CCDF) can, thus, be derived as:

$$C(S,\Omega)\text{ }=\text{ }\int D(S',\Omega)dS'\sim\Omega^{\lambda(\kappa-1)}C'(S\Omega^{\lambda})$$

where *C'*(*S*Ω^λ^) is another universal scaling function, related to *D*'(*S*Ω^λ^).

**4. Confined Plasma Model**

For nanosecond pulses with irradiances of several GW/cm^2^, the plasma induced by the laser ablation of metal targets can be described by hydrodynamic equations for the whole physical domain, where the condensed phase contributes a mass to the plasma region mainly through hydrodynamic expansion. The one-dimensional (1-D) hydrodynamics model developed earlier by Wu and Shin^S9^ can be used to calculate the plasma pressure generated during LSP in a water-confinement regime. In their model, the plasma expansion was treated as the 1-D phenomenon, because the two-dimensional (2-D) effects are important only when the laser beam diameter is very small. Wu and Shin^S10^ further demonstrated that the 1-D assumption is valid, when the laser-beam diameter is equal to or larger than 300 µm. Since the laser-beam diameter used in this work is around 1.25 mm, it is sufficient to use this 1-D model in the present work to describe the confined plasma behavior under water.

A schematic diagram of the model for the interaction of laser radiation with a target surface in the confining medium (water) is shown in Figure S4a. Initially, the metal surface is located at *z* = 0, the workpiece is at *z* < 0, and the water is in the *z* > 0 region. The sample is irradiated normally to the surface from the *z* direction. For this system, the 1-D hydrodynamic equations, governing the conservation of mass, momentum, and energy, can be expressed as^S9^

$$\frac{\partial}{\partial t}\left[\begin{matrix} \rho_{1} \\ \rho_{2} \\ \rho u \\ E+\frac{1}{2}\rho u^{2} \end{matrix}\right]+\frac{\partial}{\partial z}\left[\begin{matrix} \rho_{1}u \\ \rho_{2}u \\ \rho u^{2}+P \\ u\left(E+\frac{1}{2}\rho u^{2}+P\right) \end{matrix}\right]=\frac{\partial}{\partial z}\left[\begin{matrix} 0 \\ 0 \\ 0 \\ -q+k\frac{\partial T}{\partial z}+I \end{matrix}\right]$$

where $\rho_{1}$and $\rho_{2}$ are the densities of the metal and air (or water), respectively. $\rho$ is the total density defined as $\rho=\rho_{1}+\rho_{2}$. *u* is the velocity, *P* the pressure, *E* the volumetric internal energy, *T* the temperature, *k* the thermal conductivity, *I* the net flux in laser radiation in the z direction, and *q* the radiative heat flux in the z direction. To obtain the radiative heat flux, the radiative transfer equation needs to be solved in the diffusion approximation^S9^:

$$\begin{matrix} q=\int_{\omega} q_{\omega}d\omega,\text{ }\frac{\partial q_{\omega}}{\partial z}+ck_{\omega}ED_{\omega}=ck_{\omega}ED_{b\omega} \\ q_{\omega}=-\frac{c}{3k_{\omega}}\frac{\partial ED_{\omega}}{\partial z},\text{ }ED_{b\omega}=\frac{8\pi h\omega^{3}}{c^{3}\left(e^{h\omega/kT}-1\right)} \end{matrix}$$

in which *c* is the speed of light, *ED_ω_* and *ED_bω_* are the spectral energy densities of the plasma radiation and blackbody radiation, respectively. *k_ω_* is the absorption coefficient, and the index, ‘*ω*’, denotes the frequency-dependent quantity.

To solve the hydrodynamic equations, appropriate equations of state (EOS) must be employed. For the metal targets, the quotidian equation of state (QEOS)^S11^ is applied, which is an EOS model for the hydrodynamic simulation of high-pressure phenomena. For water, the EOS developed by Ree^S12^ is applied, which covers the density range between 2 g/m^3^ and 400 Mg/m^3^ and the temperature range between room temperature and 25 keV (2.9 × 10^8^ K) by combining theoretical codes and experimental data. Several complex phenomena are considered, such as the ionization process and the chemical equilibrium among dissociation products of water^S12^.

Figure S4b shows the plasma pressure predicted by this model for the laser beam of a 6-ns full width at half maximum (FWHM) with a 100-µm aluminum tape on the Zr-based BMG substrate. The laser-power density used in this example is 8.64 GW/cm^2^. The pulse duration for the pressure wave is approximately twice the laser pulse duration due to the confinement effect of the water layer, which has been observed by several researchers^S9,10,13,14^.

**5. Residual-Stress Calculation Procedures of Finite-Element Modeling (FEM)**

The load is modeled as a distributed pressure in ABAQUS^S1,2^, and its distribution is controlled by a user subroutine, VDLOAD^S15^. The bottom surface of the sample (the xy plane, Figures 1a and S1) is considered to be rigid.

The structural coupling between the coating-layer shock-wave pressures and the substrate structural displacements at their common surfaces (the interfaces) is accomplished with the tie constraint option in ABAQUS^S2,13^. With this constraint in the loading direction, the continuity of displacements of nodes in the interface region between the coating layer (the slave surface) and the substrate (the master surface) is ensured.

The parameters used in the calculation are listed as follows. The Young’s modulus is 82 GPa, Poisson’s ratio is 0.36, shear modulus is 35 GPa, the density is 6,810 kg/m^3^, and yield stress is 1.7 GPa^S16-20^.

**6. Assessment of Strain-Rate Effects**

In order to assess the effect of strain rate on yielding and hence the laser peened residual stress state, a strain-rate-sensitive model was also applied. A dynamic compression test of the Zr-based bulk metallic glasses (BMG) Vit-1 was reported using the split Hopkinson pressure bar^S3^. From Figure S6, it can be seen that the dynamic yield stress is close to the quasi-static value of 1.9 GPa reported in Refs. S3,S21-23 for strain rates below 1,000/s. For strain rates greater than 3,000/s, however, the compressive yield stress decreases monotonically with strain rate (strain-rate softening)^S21^. Therefore, the strain-rate softening effect on dynamic yield strength can be obtained from Figure S6.

The stress-strain curve shown in Figure 3a is considered to be in a quasi-static or static-loading condition. The stress-strain curves appropriate to different strain rates were inferred by proportionally translating the plastic part of the curve to match the different strain-rate-dependent yield stresses in Figure S6 to give the best guess curves in Figure S7. It should be noted that the above analysis assumes that the Young’s modulus is independent of strain rate. It is possible that the stress-strain curve takes a different form at high strain rates. However, in view of the lack of the available data, the curves plotted in Figure S7 are probably the best estimate available at this time. If the strain rate is higher than 4,900/s, the dynamic behavior of the BMG at 4,900/s is assumed, which will clearly underestimate the strain-rate softening effect at very high strain rates.

**Bibliography Supplementary Materials**

S1. Wu, B. & Shin, Y. C. From incident laser pulse to residual stress: a complete and self-closed model for laser shock peening. *Journal of Manufacturing Science and Engineering-Transactions of the ASME* **129**, 117-125 (2007).

S2. Cao, Y., Shin, Y. C. & Wu, B. Parametric study on single shot and overlapping laser shock peening on various metals via modeling and experiments. *Journal of Manufacturing Science and Engineering-Transactions of the ASME* **132**, 061010, 061010 (2010).

S3. Bruck, H., Rosakis, A. & Johnson, W. The dynamic compressive behavior of beryllium bearing bulk metallic glasses. *Journal of Materials Research* **11**, 503-511 (1996).

S4. Yang, C., Liu, R. P., Zhan, Z. J., Sun, L. L. & Wang, W. K. High speed impact on Zr_41_Ti_14_Cu_12.5_Ni_10_Be_22.5_ bulk metallic glass. *Materials Science and Engineering A* **426**, 298-304 (2006).

S5. Winiarski, B. *et al.* Mapping residual stress distributions at the micron scale in amorphous materials. *Metallurgical and Materials Transactions A-Physical Metallurgy and Materials Science* **41A**, 1743-1751 (2010).

S6. Dahmen, K. A., Ben-Zion, Y. & Uhl, J. T. Micromechanical model for deformation in solids with universal predictions for stress-strain curves and slip avalanches. *Physical Review Letters* **102**, 175501 (2009).

S7. Dahmen, K. A., Ben-Zion, Y. & Uhl, J. T. A simple analytic theory for the statistics of avalanches in sheared granular materials. *Nature Physics* **7**, 554-557 (2011).

S8. Antonaglia, J. *et al.* Tuned critical avalanche scaling in bulk metallic glasses. *Scientific Reports* **4**, 4382 (2014).

S9. Wu, B. & Shin, Y. C. A one-dimensional hydrodynamic model for pressures near the coating-water interface during laser shock peening. *Journal of Applied Physics* **101**, 023510-023515 (2007).

S10. Wu, B. & Shin, Y. C. Two dimensional hydrodynamic simulation of high pressures induced by high-power nanosecond laser-matter interactions under water. *Journal of Applied Physics* **101**, 103514 (2007).

S11. More, R. M., Warren, K. H., Young, D. A. & Zimmerman, G. B. A new quotidian equation of state (QEOS) for hot dense matter. *Physics of Fluids* **31**, 3059-3078 (1988).

S12. Ree, F. Equation of State of Water. (California Univ., Livermore USA, Lawrence Livermore Lab., 1976).

S13. Berthe, L., Fabbro, R., Peyre, P., Tollier, L. & Bartnicki, E. Shock waves from a water-confined laser-generated plasma. *Journal of Applied Physics* **82**, 2826-2832 (1997).

S14. Wu, B. & Shin, Y. C. A self-closed thermal model for laser shock peening under the water confinement regime configuration and comparisons to experiments. *Journal of Applied Physics* **97**, 113517, 113517 (2005).

S15. Systèmes, D. in *ABAQUS v6.9 Documentation* (2009).

S16. Lin, X. H., Johnson, W. L. & Rhim, W. K. Effect of oxygen impurities on crystallization on undercooled bulk glass forming Zr-Ti-Cu-Ni-Al alloy. *Mater. Trans. JIM* **38**, 473-477 (1997).

S17. Nieh, T. G., Wadsworth, J., Liu, C. T., Ohkubo, T. & Hirotsu, Y. Plasticity and structural instability in a bulk metallic glass deformed in the supercooled liquid region. *Acta Materialia* **49**, 2887-2896 (2001).

S18. Peter, W. H., Buchanan, R. A., Liu, C. T. & Liaw, P. K. The fatigue behavior of a Zirconium-based bulk metallic glass in vacuum and air. *Journal of Non-crystalline Solids* **317**, 187-192 (2003).

S19. Morrison, M. L. *et al.* Four-point-bending-fatigue behavior of the Zr-based Vitreloy 105 bulk metallic glass. *Materials Science and Engineering a-Structural Materials Properties Microstructure and Processing* **467**, 190-197 (2007).

S20. Tang, C. G., Li, Y. & Zeng, K. Y. Characterization of mechanical properties of a Zr-based metallic glass by indentation techniques. *Materials Science and Engineering A-Structural Materials Properties Microstructure and Processing* **384**, 215-223 (2004).

S21. Lu, J., Ravichandran, G. & Johnson, W. Deformation behavior of the Zr_41. 2_Ti_13. 8_Cu_12. 5_Ni_10_Be_22. 5_ bulk metallic glass over a wide range of strain-rates and temperatures. *Acta Materialia* **51**, 3429-3443 (2003).

S22. Yang, Q., Mota, A. & Ortiz, M. A finite-deformation constitutive model of bulk metallic glass plasticity. *Computational Mechanics* **37**, 194-204 (2006).

S23. Yokoyama, Y., Yamasaki, T., Nishijima, M. & Inoue, A. Drastic increase in the toughness of structural relaxed hypereutectic Zr_59_Cu_31_Al_10_ bulk glassy alloy. *Materials Transactions* **48**, 1276-1281 (2007).
